# Whose nose does not know? Demographical characterization of people unaware of anosmia

**DOI:** 10.1007/s00405-019-05414-8

**Published:** 2019-04-15

**Authors:** Anna Oleszkiewicz, Thomas Hummel

**Affiliations:** 1 Smell and Taste Center, Department of Otorhinolaryngology, TU Dresden, Fetscherstrasse 74, 01307 Dresden, Germany; 2 Institute of Psychology, University of Wroclaw, ul. Dawda 1, 50527 Wroclaw, Poland

**Keywords:** Olfaction, Anosmia, Normosmia, Psychology, Smell

## Abstract

**Purpose:**

For functionally anosmic subjects, the sense of smell is basically useless in daily activities—they are unlikely to detect the threatening smell of rotten food, gas or smoke, or to enjoy the flavor of food or the smell of perfumes. Although this appears very distressing, functionally anosmic subjects in our sample seemed not to be aware or bothered with impaired olfaction and enrolled for the study targeted to people with a normal sense of smell.

**Methods:**

In the large sample of 9139 subjects who declared themselves to have a normal sense of smell, we have retrospectively found a notable proportion of scores indicating functional anosmia.

**Results:**

When we look at the overall Sniffin’ Sticks score, 0.45% of the sample was functionally anosmic and this fraction increased to 3.4% when the identification score of 8 points and below was used. We present demographical information of those subjects, who despite their inability to use smell in daily life, consider themselves healthy.

**Conclusions:**

Data offer a new perspective on the importance of olfaction in daily life and supports the notion about the importance of using screening tools in clinical practice.

## Introduction

The updated Sniffin’ Sticks normative dataset [1] revealed a notable proportion of subjects whose score indicates functional anosmia despite the fact that all of them reported normal olfactory function.

The sample we utilized included only subjects who considered themselves healthy and reported a normal sense of smell. Importantly, the cohort we have been working with was relatively young (4928 females aged 5–96 years (*M* = 31.8, SD = 18.9 years) and 4211 males aged 5–91 years (*M* = 30.7, SD = 17.7 years) with 50% of the sample being younger than 25 years and 95% of the sample being younger than 68 years. Based on the combined score of odor threshold, odor discrimination and odor identification subtests, we found 0.45% subjects with results of 16 points and below suggesting functional anosmia (*n* = 15; 5 females aged between 79–90 years *M* = 84.2, SD = 4.1 years, and 10 males aged 44–89 years, *M* = 71.3, SD = 14.7 years of the total number of 3356 individuals with the complete TDI score). This fraction increased to 3.4% when the identification score of 8 points and below was used to define functional anosmia (*n* = 310 of the total number of 8348 individuals with a score in the odor identification task only [note: throughout the text functional anosmia is referred to as “anosmia”]). For all these subjects, the sense of smell was basically useless in daily activities—they are unlikely to detect the threatening smell of rotten food, gas or smoke, or to enjoy the flavor of food or the smell of perfumes. But still they seemed not to be bothered with it and reported a normal sense of smell.

The criterion for functional anosmia based on the odor identification test only revealed that decreased odor identification ability was more frequent among females, who were significantly older than men, *t *(308) = 2.53, *p* = 0.01—see Table 1.

**Table 1:** Demographical characteristic of males and females being unaware of their functional anosmia—based on identification score of 8 points and below

	Female	Male
*N*	190	120
Age *M* [in years]	55.7	46.7
Age SD	31.6	28.6
Age min	6	5
Age max	96	91
Smokers	3	8

**Fig. 1:**
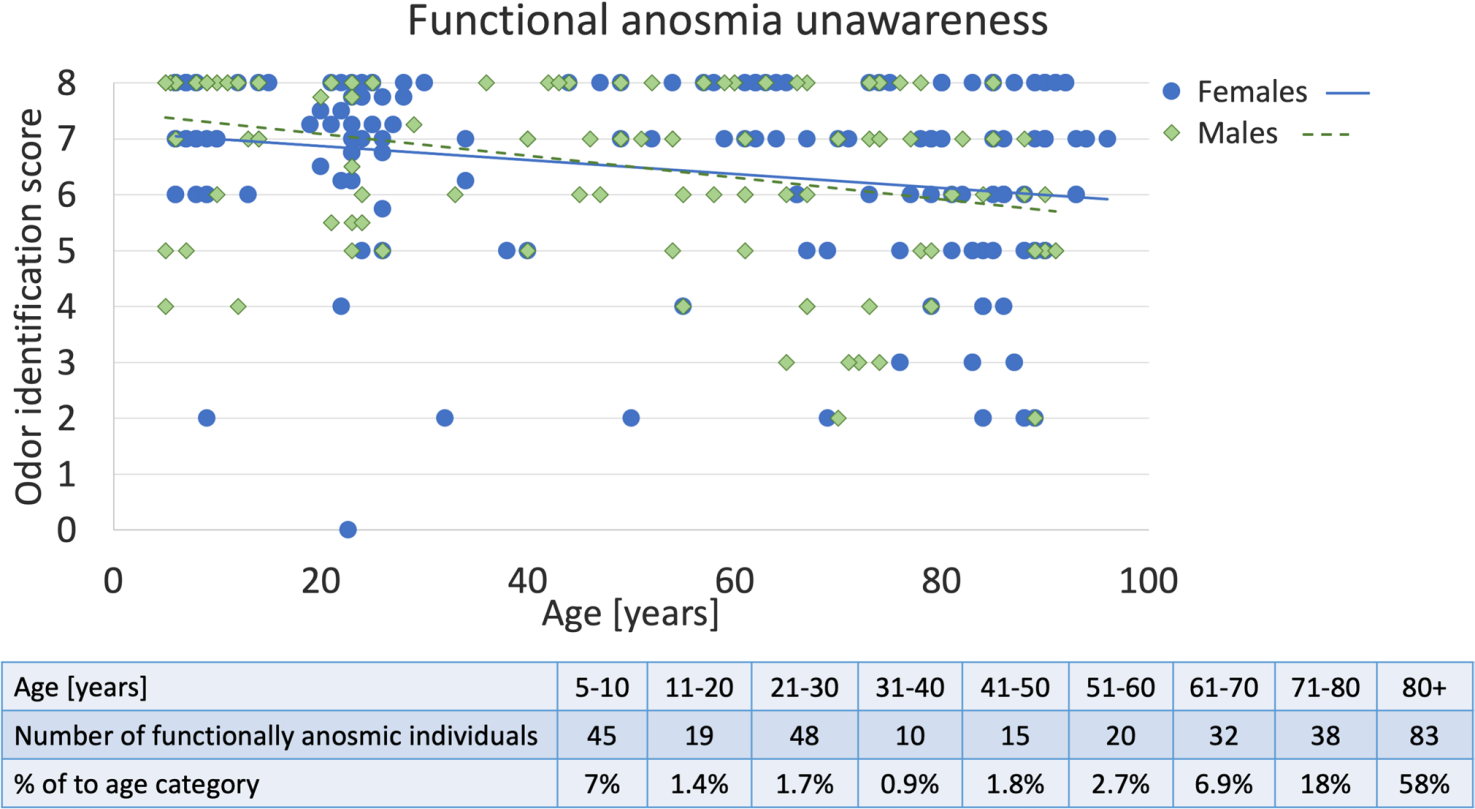
Prevalence of functional anosmia among male and female subjects declaring no olfactory impairment. Table at the bottom presents the number and fraction of individuals unaware of functional anosmia across the nine age groups

Figure 1 presents the distribution of scores plotted against the age of male and female subjects. The issue of anosmia unawareness does not simply reflect a parabolic distribution—the prevalence of identification scores of 8 points and below among males and females was highest in youngest and oldest age groups, yet it is notable also in the middle-aged subjects who normally are at the height of their olfactory capabilities [2, 3]—see Fig. 1.

Data further supports the notion that despite self-reported normal olfaction, severe olfactory impairment is present in a considerable proportion of subjects of all ages [4–6]. It can be argued that females generally outperform males in olfactory tasks [7–9] and that female’s behavior and decisions are influenced by odors to a larger extent than male’s [10, 11], yet further investigation targeted into variables explaining occurrence of unconscious functional anosmia is required.

Based on identification score of 8 points or less, anosmia was found to be more frequent in females than in males. In addition, an impaired ability to recognize odors also occurs among younger subjects (i.e., children, adolescents and young adults) as compared to deteriorated overall olfactory function (TDI score of 16 points and below). Various medical (e.g., history of infections, head trauma) and psychological conditions including cognitive deterioration resulting from aging and disrupted emotional processes like depression [5, 12] may explain the decreased ability to recognize commonly known odors. Of note, among anosmic subjects only very few were smokers. Although negatively related with overall olfactory performance [13], cigarette smoking is not common among subjects unaware of their functional anosmia [14].

Our findings support the argument for using screening tools for olfactory assessment in clinical practice. Surprisingly, a number of subjects consider themselves healthy and seem not to be bothered by the practical inability to smell. Thus, there is a considerable risk of false-negative diagnosis, especially in older people, when the diagnosis is based primarily on the medical interview including self-reported olfactory performance. Putting aside possible etiology, the absence of the sense of smell can cause a deterioration in the quality of life [15–18] which patients might have difficulties to attribute to a certain health problem. As a consequence, they will not aim to undertake an adequate treatment. The latter argument should be of particular interest for psychologists and psychiatrists.

Scientists have no doubts that olfaction is of great importance for human interpersonal relationships and mating [19–22], regulation of emotions and behavior [23–25], learning and cognitive processes [26–28]. Functionality of olfactory system is profoundly associated with Parkinson’s disease [29–31], Alzheimer’s disease [32–34], and depression [35, 36]. Presented evidence provokes a speculation that olfaction does not affect professional lives in most people and is not disabling itself. For instance, some patients with congenital anosmia do not notice the absence of smell in their lives and do not complain about it. Elderly subjects, whose sense of smell deteriorates gradually as a function of age, also find a way to cope with the inability to perceive odors. These observed examples speak against our common thinking of the sense of smell as a necessary condition for good health and satisfying life and urges to put the meaning of human olfaction into perspective [37]. Counterarguments could have their source in the more detailed social and psychosomatic characterization of people unaware or not bothered by anosmia and those who cannot smell yet are still convinced they can.

An important limitation of the current investigation is an uncontrolled comprehension and attention paid to the task by the subjects that could potentially translate into a low score. Although the Sniffin’ Sticks examination was performed by well-trained staff at the Smell and Taste Center, assuring experience, clear instructions and additional explanations to the subjects if needed, we cannot rule out that some of the subjects with very low scores had difficulties to comprehend the task. However, the identification task being the simplest of the three Sniffin’ Sticks tasks and usually the most engaging produced 3.4% of scores in the range of functional anosmia in subjects of various age.
